# Psychological Outcomes of Cognitive Impairment and Dementia: Anxiety, Perceived Stress and Depressive Symptoms

**DOI:** 10.1002/alz70861_109004

**Published:** 2025-12-23

**Authors:** Ayse Malatyali, Rui Xie, Eunice Ojo, Tom Cidav, Ladda Thiamwong, Breno S Diniz

**Affiliations:** ^1^ University of Central Florida, 12201 Research Parkway, Suite 300 Orlando, FL USA; ^2^ University of Central Florida, Orlando, FL USA; ^3^ University of Central Florida, Orlando, FL, 32826, FL USA; ^4^ U.S. Department of Veterans Affairs, Philadelphia, PA USA; ^5^ University of Connecticut Health Center, Frmington, CT USA

## Abstract

**Background:**

Psychological conditions are significant health concerns among older adults. However, limited evidence exists on how different cognition levels are associated with various psychological conditions. This study examines the associations of cognition levels with anxiety, perceived stress, and depressive symptoms among older adults.

**Method:**

Using logistic regression and generalized linear models, we analyzed cross‐sectional data from 2,730 participants (age ≥ 65) in the Health and Retirement Study (HRS) 2020 interviews. Cognition was assessed with the 27‐point HRS cognition scale; Psychological outcomes were measured with the 5‐item Beck Anxiety Inventory, Perceived Stress Scale, and the Center for Epidemiological Studies Depression Scale.

**Result:**

Cognitive impairment and dementia were strongly associated with the burden of psychological conditions. Individuals with cognitive impairment have 39% higher odds of anxiety, 0.99‐point higher perceived stress, and 0.33‐point higher depressive symptoms than individuals with normal cognition. Individuals with dementia were 91% more likely to experience anxiety, have 2.5‐point higher perceived stress, and 0.37‐point higher depressive symptoms than those with normal cognition. Women had significantly higher rates of anxiety, stress, and depressive symptoms than men. Hispanic individuals had higher anxiety and higher depressive symptoms than non‐Hispanic individuals, while their perceived stress scores were not significantly different. Multimorbidity with two or more chronic conditions, functional difficulty, and lower income were also significant predictors of poorer psychological health.

**Conclusion:**

Findings highlight the disproportionate burden of psychological conditions for older adults with cognitive impairment and dementia, emphasizing the need for tailored intervention to improve psychological well‐being in the continuum of Alzheimer's Disease and Related Dementias.

